# Regressions during Reading

**DOI:** 10.3390/vision3030035

**Published:** 2019-07-09

**Authors:** Albrecht W. Inhoff, Andrew Kim, Ralph Radach

**Affiliations:** 1 Department of Psychology, Binghamton University, Binghamton, NY 13902, USA; 2 Department of Psychology, Bergische Universitaet, 42103 Wuppertal, Germany

**Keywords:** reading, eye movements, regressions, individual differences

## Abstract

Readers occasionally move their eyes to prior text. We distinguish two types of these movements (regressions). One type consists of relatively large regressions that seek to re-process prior text and to revise represented linguistic content to improve comprehension. The other consists of relatively small regressions that seek to correct inaccurate or premature oculomotor programming to improve visual word recognition. Large regressions are guided by spatial and linguistic knowledge, while small regressions appear to be exclusively guided by knowledge of spatial location. There are substantial individual differences in the use of regressions, and college-level readers often do not regress even when this would improve sentence comprehension.

## 1. Introduction

Visual text consists of symbols that are spatially ordered along horizontal rows or vertical columns. Typically, a large number of symbols is visible concurrently, and they are visible for an extended period until a screen is changed or a page is turned. Speech, by contrast, consists of a temporally ordered sequence of acoustic symbols, and only a very limited amount of linguistic information is available at each point in time. The extraction of linguistic information during reading thus requires modality-specific skills. With reading, these skills include the programming of eye movements that position the eyes at—or near—individual words, as high acuity vision is confined to a relatively small retinal area: The fovea and adjoining parafovea. The spatial targeting of eye movement programming needs to be coordinated with linguistic processing, so that high acuity vision is moved to words when their identification becomes relevant for text comprehension. Most eye movements (saccades) progress with word order, from left-to-right for Roman and modern Chinese script, right-to-left for Hebrew and Arabic script, and also from top to bottom with traditional Chinese script. 

A distinct subset of saccades, 5–20%, however, moves the eyes in a direction that is opposite to word order [1,2,3,4]. Kolers [5] noted that these reversals of saccade direction do not interfere with reading comprehension, and Rayner [4] suggested that they are responses to reading difficulties. The current review extends prior overviews in several aspects: We argue that there are two distinct types of regressions, that they serve distinct functions, and that their targeting is controlled by somewhat different types of representations. We also review individual differences in the use of regressions and consider potential implications for the teaching of reading. 

## 2. The Spatial Targeting of Regressions 

Corpus analyses show that regressions differ substantially in size [2,3]. They can reposition the eyes closer to the beginning of the fixated word, move the eyes to an adjacent prior word, or move across several prior words. When multi-line text is read, regressions can even cross one or more lines. The implications of these substantial differences in the size of regressions have remained unexplored. 

### 2.1. Large Regressions 

The literature has primarily focused on “large” regressions that traverse across more than one prior word [4]. Though words that are the target of a large regression are viewed out of order, Kolers [5] suggested that their viewing does not interfere with text comprehension because grammatical word order is determined by words’ spatial location. Kennedy [6] (see also [7,8]) elaborated on this view. In his conception, identified words are represented in conjunction with a spatial tag that indexes their location on a line, or their relation to a spatial reference frame. This spatial indexing is assumed to be part and parcel of visual word recognition and to occur automatically. When processing difficulties can be linked to a prior word or a prior text segment, the indexes of corresponding text are retrieved and used for regression targeting. 

In this scheme, spatial indexes assume two useful functions: They guide a regression to a previously read text segment, the hypothesized source of a processing difficulty, and they correct the ensuing mismatch between the temporal order with which words are viewed and grammatical word order. If, for instance, a reader executes a regression from word location seven to word location three on a line of text, then the spatial index of the regressed-to word informs the reader that word three will be re-inspected. 

Kennedy’s experimental work [8,9] appeared to provide compelling support for this hypothesis. Since the occurrence and targeting of large regressions cannot be controlled under normal reading conditions, a probe classification task was devised that resulted in the likely execution of a regression with controlled starting and ending points. Specifically, sentences were constructed that contained a target word at a specific location. Participants were asked to read the sentence, and then to view a probe word that was shown to the right of the sentence. The task was to determine whether the probe had appeared in the sentence (see sample sentences 1a and 1b). In the original study [9], the probe was either identical to the target word in the sentence (1a) or a semantically related word (1b).

1a. The man was looking for a spade in the shed next to the barn. Spade
1b. The man was looking for a spade in the shed next to the barn. Shovel

Under these conditions, regressions toward the target occurred on approximately a quarter of trials. These regressions were remarkably accurate, as they positioned the eyes consistently near the center of the target word, *spade* in the example, irrespective of probe-target distance. Consistent with the spatial coding hypothesis, readers were assumed to use the probe to find the related spatial index in the sentence, and to use it for the targeting of the regression. 

#### Shortcomings of the Spatial Coding Hypothesis 

By design, the spatial location of words on a line of text is confounded with their grammatical order. When this relationship is removed, by presenting the words of a sentence at arbitrary screen locations, knowledge of a word’s spatial location is poor and short-lived [10]. This finding is difficult to reconcile with the spatial coding hypothesis. The hypothesis was also challenged by additional experiments that examined the accuracy of regressions whose start and end points are experimentally controlled. Although the size of these regressions increased with the distance of the regression target, the reaching of these targets was error prone, in particular, when the target was far. In this case, regressions often missed a designated word location by several words [11,12,13]. 

In Weger and Inhoff [11], the regression error for text that extended across two lines increased not only with spatial (horizontal and vertical) distance but also with the number of words that intervened between the starting and end points of a regression. Furthermore, readers executed additional, search-like saccades that moved the eyes onto the target, when the initial regression to the designated word location was inaccurate. Linguistic information thus influenced the accuracy of regression targeting. Related work by Guerard and collaborators showed larger regression errors, in particular for far targets, when the reading task was accompanied by an articulatory suppression task that involved the repeated articulation of the consonant sequence “ABCD” at a pace of two second per letter [14,15]. These results suggest that phonological working memory may be involved in the specification of far locations, as articulatory suppression was likely to interfere with the representation of phonological forms in working memory. 

Rather than retrieving the spatial index of a designated regression target, readers may use the working memory representation of previously recognized words (and linguistic knowledge) to estimate how far back a prior word had been read, and this could be used to be used to estimate a target’s spatial coordinates [15]. The difficulty of this estimate may increase with the number of words that intervene between the fixated word and with the target’s spatial distance. 

### 2.2. Small Regressions

The vast majority of regressions reposition the eyes within a word or moves the eyes to the spatially adjacent prior word. Several findings indicate that the programming of these small regressions differs from the programming of large regressions. They often follow an oculomotor targeting error [2,3], and regressions up to 10 letter spaces move the eyes with a remarkable degree of accuracy to the center of the preceding word [16], that is, they differ from similar size forward-directed saccades in that they are not subject to systematic targeting errors. Presumably, this occurs because the spatial properties of the regression target are still represented in working memory [17]. The results of a recently completed study [18] are consistent with this view. Readers with upper quartile spatial working memory scores executed more accurate regressions than readers with lower quartile scores when the regression target was near but not when it was far. 

## 3. The Function of Regressions 

Text remains generally available after it has been read, and it can thus be used like an external storage device. If a processing impasse can be related to a previously read text segment, a reader can re-view it with a flick of the eyes and re-read the segment. Re-processing may reveal whether and why represented linguistic content differed from the actual text. This can be used to validate or update the representation of corresponding linguistic content. In the case of small regressions, two reasons for making movements against the direction of reading can be specified. First, there are cases when the eyes land on a word that was not the intended recipient of a saccade, especially when a word was accidentally “skipped”. Second, there can be a need to return to a preceding word when it was not fully processed during fixation [19]. These possibilities will be explored later, but for now the focus is on long range regression in the service of comprehension. 

### 3.1. Regressions for Text Comprehension 

The “re-viewing for reprocessing” hypothesis [5,20] is the prevailing account for the execution of large regressions. It maintains that a previously read word or sentence segment become the target of a regression when their represented meaning (or grammatical role) disagrees with subsequently read linguistic content. For instance, the heterophonic homograph “bass” in the sentence “The fisherman was looking for the bass” should become the target of a regression when the representation of “bass” as a type of fish disagrees with the remainder of the sentence, “that the guitarist had dropped in the lake” [21], which implies that “bass” refers to a type of musical instrument. Similarly, syntactic parsing may go wrong in sentences with garden path constructions. In the sentence, “Because Ed drinks vodka is everywhere in the house”, comprehension is likely to be break down when the words “is everywhere” are identified. This occurs because “vodka” is preferentially parsed as the object of “Ed drinks” rather than as the subject of “is everywhere”. Readers may regress to reprocess prior text in order to correct the parsing error. 

One alternative to the reprocessing hypothesis is that large regressions are not aimed at a particular text location [22]. Instead, they benefit processing primarily because they provide additional processing time. Consistent with this view, detailed analyses of the starting and end locations of regressions in response to syntactic garden pathing showed that regressions did not consistently move the eyes to the source of the processing difficulty (the misparsed sentence segment). In other experiments, readers occasionally regressed toward a previously read sentence segment even when it was no longer visible on a screen for reviewing and reprocessing [8,23]. 

These eye movements to blank screen locations are also consistent with another alternative, according to which the primary function of regressions is to enhance retrieval from working memory. Laeng and Teodorescu [24] showed that the sequence of saccades during the generation of a mental image of a previously viewed visual pattern was similar to the sequence of saccades when the visual pattern was originally viewed, as if moving the eyes to previously viewed locations supported content-specific imagery. Moreover, memory for a previously viewed visual pattern was more accurate when participants could move their eyes during the memory task than when their eyes had to remain fixated. Presumably, this occurred because saccades to the location of previously depicted objects supported the retrieval of objects that were viewed at those locations. 

To distinguish between these accounts, Booth and Weger [25] constructed sentences with endings that required knowledge of a critical target word that had occurred earlier in the sentence, the assumption being that readers would regress to the target to validate it or revise its representation. In Experiment 3, the target was either unchanged or it was replaced with another word when the eyes regressed to the target location (e.g., the word “driver” was replaced with the word “dancer”). Sentence reading was followed by a multiple choice task with three alternatives, one that referred to the meaning of the originally presented target word, another that referred to the regression-contingent substitute, and yet another to a word that did not appear in the sentence (the target and replacement word were not presented in the multiple choice task). In the absence of regressions, or when regressions did not land on the target word location, the target-related meaning was selected on 70% of the trials. Conversely, a meaning related to the substituted word was selected on 68% of the trials when a regression to the target location had resulted in the “re-viewing” of the replacement word at that location. This indicates that regressions were used to re-process the word. and that the meaning of the original word was replaced with the meaning of the re-processed substitute. 

Similar results emerged from the key condition of a follow-up study [26] in which a target word, e.g., “house” was inconsistent with the meaning of a subsequent verb, e.g., “ridden”. A regression to the target either left it unchanged or replaced it with a congruent word, “horse” in the example. Substantially less re-viewing time was spent on the target when it was changed to a congruent word, presumably because the replaced target offered a better contextual fit. Consistent with the reprocessing hypothesis, these findings demonstrate that readers re-processed the word that was reached with a regression, and that the outcome of re-processing was used to update represented sentence content. 

Other studies also show that large regressions can improve comprehension. Using eye-movement-contingent display changes to manipulate the visibility of text to the left of a fixation, so that it was either masked or re-readable after a regression, Schotter et al. [27] showed that sentences with garden path constructions were understood more successfully when readers re-read text after a regression. Readers also appear to use regressions to correct word identification errors [28], and to fill in “missed” sentence and story parts that were viewed during episodes of mind wandering and attentional lapses [29].

#### 3.1.1. The Frequency of Regression Usage 

Processing difficulties increase the frequency of regressions. When readers read sentences with contextually incongruent and congruent words [26], regressions out of the end of the sentence occurred on 60% of the trials in which a previously read target word was incongruent with a subsequently read verb, and on 20% of the trials in which the two words were congruent. Surprisingly, the frequencies of outgoing regressions differed little when the mismatching verb itself, “ridden” in the prior example, was fixated, which had an outgoing regression rate of 25%, when it was incongruent with the target [house -> ridden], and 18%, when it was congruent [horse -> ridden]. 

Using a similar approach, we examined the rate of regressions that moved the eyes onto the source of a presumed processing difficulty [28]. The materials were constructed so that controlled target words would be the source of a processing difficulty in an incongruent condition but not in a congruent condition. For instance, in the sentence “The midwife thought that the birch was successful despite the mother’s concerns”, the target “birch” is incongruent with the subsequent sentence context. A visually similar word, “birth”, was the target for the same sentence frame in the congruent condition (with a different sentence frame “birch” was congruent and “birth” incongruent). With this approach, regressions to incongruent targets were approximately twice as common as regressions to congruent targets. However, regressions to incongruent targets were not routinely executed. Across experiments, the highest rate of regressions onto incongruent targets was 32%. 

Similarly, a detailed examination of regression usage during the reading of sentences with garden-path constructions [22] (see also [30]) showed that parsing difficulties were not routinely responded to with a regression. In the study, readers regressed out of the breakdown region of garden path sentences on approximately 25% of trials, when the region was on the same line as the misparsed phrase, and on approximately 17% of trials, when the breakdown region and the misparsed phrase were on different lines. Moreover, regressions out of the breakdown region were not preferentially directed at the misparsed phrase, and, as noted earlier, regressions out of verbs (“ridden”) were approximately as common when they were congruent with a prior target as when they were incongruent. 

Why were comprehension difficulties not routinely and/or not immediately responded to with a regression? According to Folk and Morris [31] (see also [32]) this may occur because readers prefer to use the represented forms of previously read words (in phonological working memory) for the resolution of processing difficulties. 

In these experiments, prior context did not constrain the meaning of lexically ambiguous target words with biased meanings. Subsequent text was, however, constructed so that it implied the subordinate meaning of a target, that is, it disagreed with the likely selection of the preferred target meaning. In two pertinent conditions, targets were either homographic homophones (e.g., “bank”) or homographic heterophones (“tear”). Homophones and their controls differed in that readers spent more time viewing post-homophone context. With heterophones, by contrast, readers increased the rate of regressions from post-target context to targets (see also [21]). According to Folk and Morris [31], homophone representations could be corrected with a retrieval of working memory (WM) forms, as represented phonological target forms could be used to retrieve a context-consistent subordinate meaning. Regressions were executed when the retrieval of phonological forms did not provide access to an alternative meaning for the resolution of the integration difficulty. To obtain a different phonological form, homographic heterophones had to be reviewed with a regression. 

#### 3.1.2. The Timeline of Regression Programming 

The timeline of eye movement programming appears to be similar when large regressions are executed for the re-viewing and reprocessing of text and when forward-directed saccades are executed for the identification of upcoming words. A comparison of regressive and progressive saccades out of the ‘breakdown region’ of sentences with garden path constructions [30] showed that the mean fixation duration prior to the execution of a saccade was approximately 235 ms, irrespective of whether the outgoing saccade regressed or progressed in the sentence when several words were visible to the right and left of the fixated breakdown region. Regressions and progressions differed, however, in size, with a mean of 18 character spaces for regressions and of eight character spaces for progressions. 

The programming of large regressions and of forward-directed saccades could have similar timelines because their programming occurs in response to information that is extracted from the fixated segment of text. If the extracted information is incongruent with represented content (and when use of working memory did not resolve the processing difficulty), readers may decide to regress to reprocess prior text. If the extracted information is congruent, readers may move their eyes forward to identify upcoming words. 

### 3.2. Small Regressions 

Corpus analyses show that regressions are, on average, smaller than forward-directed saccades [2,11,33], extending typically across one to three letter spaces. The viewing duration preceding these regressions is typically short [34], as if these regressions corrected a poor viewing location [34,35] that was not well suited for word recognition [36]. 

Small regressions often move the eyes from the ending of a word toward the center or the beginning to improve its recognition. Other small regressions move the eyes from a fixated word to the immediately preceding word, and these inter-word regressions are particularly common when the prior word has not been fixated (i.e., when it was skipped) [32]. Similar to regressions within a word, post-skip regressions could be used to obtain a better viewing location for the identification of skipped word. 

Although word skipping can occur when an upcoming word is recognized before a saccade to it is committed to execution [33,37], corpus analyses not only indicate that readers generally direct the eyes at the center of an upcoming word [34,37,38], but also that this location is often missed. These presumed targeting errors are attributed to a range error that biases the size of executed saccades toward a default (mean) saccade size, and an additional random error. These findings imply that some word skipping is due to oculomotor targeting errors, and computational simulations (e.g., [39]) are consistent with that. An erroneous skipping of a word is likely to impair its recognition, and this could be corrected with a small regression to the skipped word. 

Readers may also execute small regressions to correct oculomotor timing errors. That is, the eyes may leave a fixated word before its processing has been completed. Akin to a spatial targeting error, premature saccades will position the eyes off a to-be-processed word, and this divergence can be corrected with a small regression [35,40].

#### 3.2.1. Frequency of Small Corrective Regressions 

It appears plausible to assume that oculomotor targeting errors would be corrected routinely when they impair word recognition, and this appears to be the default assumption in current conceptions of eye movement control during reading [35,41]. However, corpus analyses cannot examine the link between word skipping, recognition failures, and corrective regressions, as the success of visual word recognition is not self-evident. Even when the recognition of a skipped word failed, a reader could move the eyes forward after skipping and perhaps use upcoming text to infer the identity of the skipped word. To illuminate the link between skipping, word recognition failure, and corrective regressions, we examined the occurrence of regressions after the skipping of words that could not be recognized prior to skipping. 

In the experiment (Experiment 1) [42], the display of three-letter target words (e.g., “tax”) was manipulated so that they were either visible (pre-viewable) throughout sentence reading or masked with a length-matched string of visually dissimilar random letters (“gfj”). The mask occupied the target location until the eyes reached a display-change boundary, the blank space preceding the target location. A saccade to the right of the boundary replaced the random letter mask with the target word, and the intact target was always shown when the eyes landed on the target location or to the right of it. Since targets were relatively short words, they were liable to skipping, either because pre-viewable targets were recognized before a target-reaching saccade was committed to execution, or due to erroneous oculomotor overshoot (irrespective of whether the target had been previewed or masked). 

Examination of boundary-crossing saccades showed that the skipping of the target area was relatively common (31%; 687 of 2167 trials). This rate is similar to the skipping rate of three-letter words in other studies (e.g., [43]). Slightly more than half of the target skips (58%; 395 of 687) occurred in the target visible condition; on the remaining skipping trials, a random letter mask, such as “gfj”, was skipped, presumably due to oculomotor targeting error. When this occurred, the erroneous fixation of the post-target word should have impeded target word recognition, and regressions from post-target words to target words should have occurred routinely. 

**Figure 1:**
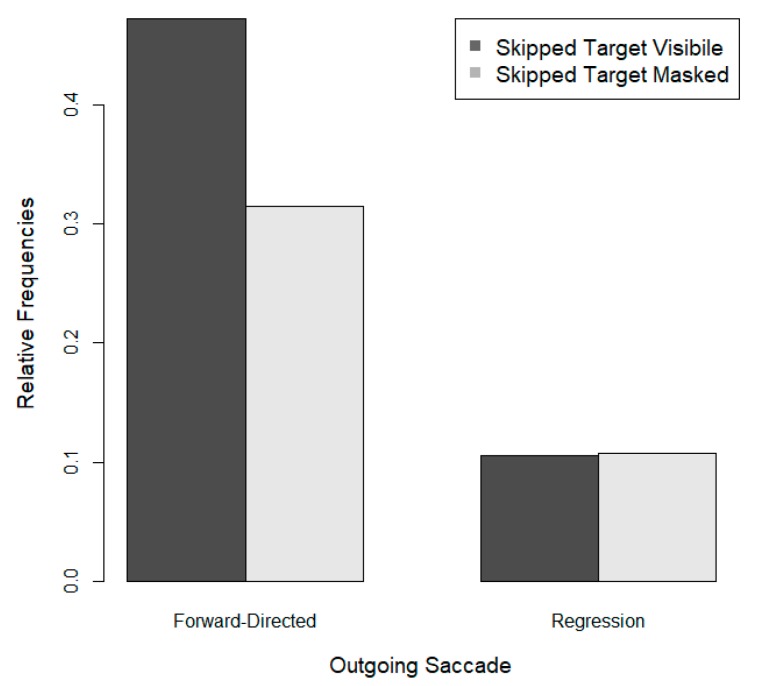
The relative frequencies of saccades out of post-target words after the preceding three-letter target area had been skipped. Relative frequencies are shown as a function of the visibility of the target word prior to the skipping of the target area and as a function of the direction of the outgoing saccade (relative frequencies add up to 1).

The relative frequency of saccades from the post-target word to the target after the skipping of previously masked and visible target is shown as a function of the direction of the outgoing saccade in Figure 1. It is evident that the skipping of a masked target was not routinely corrected with a regression. To the contrary, forward-directed saccades out of the post-target word were approximately three times as common as regressions back to the target after masked target skipping. Moreover, regressions to skipped masked targets were as common as regressions to skipped visible targets. These findings imply that erroneous oculomotor targeting is not routinely corrected with a regression. 

**Figure 2:**
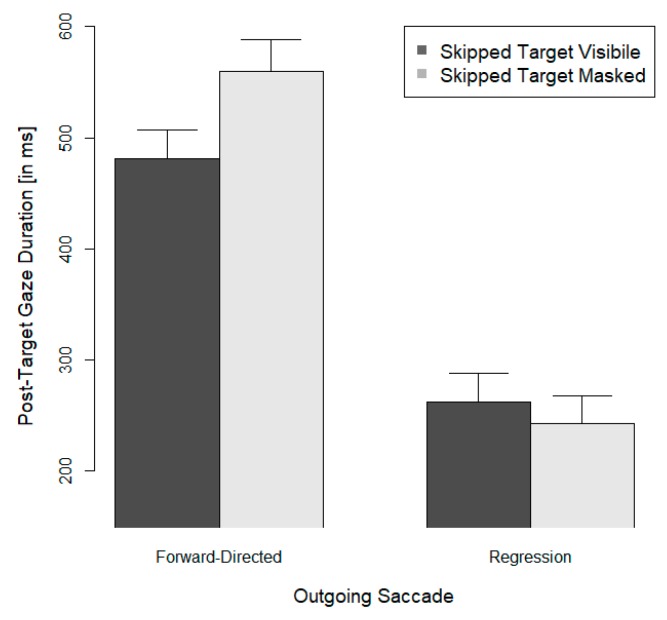
Mean gaze durations and their standard errors for post-target words when the preceding target word had been skipped. Means are shown as a function of the visibility of the target word prior to skipping and of the direction of the saccade out of the fixated post-target word.

An analysis of the viewing time (gaze) for post-target words after target skipping (Figure 2) showed that the skipping of a target resulted in relatively long post-target viewing durations (approximately half a second) when readers moved out of the post-target word with a forward-directed saccade, and this occurred, in particular, when a masked target had been skipped. The relatively long post-target viewing duration when readers exited with a forward-directed saccade suggests that readers sought to identify the skipped target while the post-target word was fixated. This was particularly difficult when the skipped target word had been masked. 

Together, these analyses indicate that oculomotor targeting errors are relatively common, and that they increase the difficulty of word recognition. Critically, they also show that the increase in word recognition difficulty is not routinely responded to with a regression. 

#### 3.2.2. The Time Course for the Programming of Corrective Regressions

Figure 2 also shows relatively short post-target viewing durations (less than a quarter of a second) when a regression to the skipped target was executed. The large size of the saccade direction effect suggests that the extraction of post-target information was short-circuited prior to the execution of a corrective regression to a skipped target. To determine whether useful information was extracted from a post-target word prior the execution of a regression to a skipped target, we analyzed post-target viewing durations as a function of a skipped target’s visibility prior to boundary crossing and of the information that could be extracted at the onset of post-target viewing (Experiment 2) [42]. As in Experiment 1, the visibility of a target prior to boundary crossing was manipulated so that the three-letter target was either visible or masked before the eyes crossed the boundary. Again, the intact target was shown when the eyes landed on it or skipped it. In addition, Experiment 2 manipulated the visibility of the post-target word after target skipping. The post-target word was now either visible (in upper case) at the onset of the post-skip fixation or its presentation was delayed by 50 ms (a string of random letters occupied the post-target position for 50 ms in this case). In both viewing conditions, the post-target word was visible in lower case 50 ms after the onset of its fixation. If readers sought to obtain linguistic information from the fixated post-target word prior to regression programming, then fixation durations preceding the regression should be longer when a post-target word’s onset was delayed.

**Figure 3:**
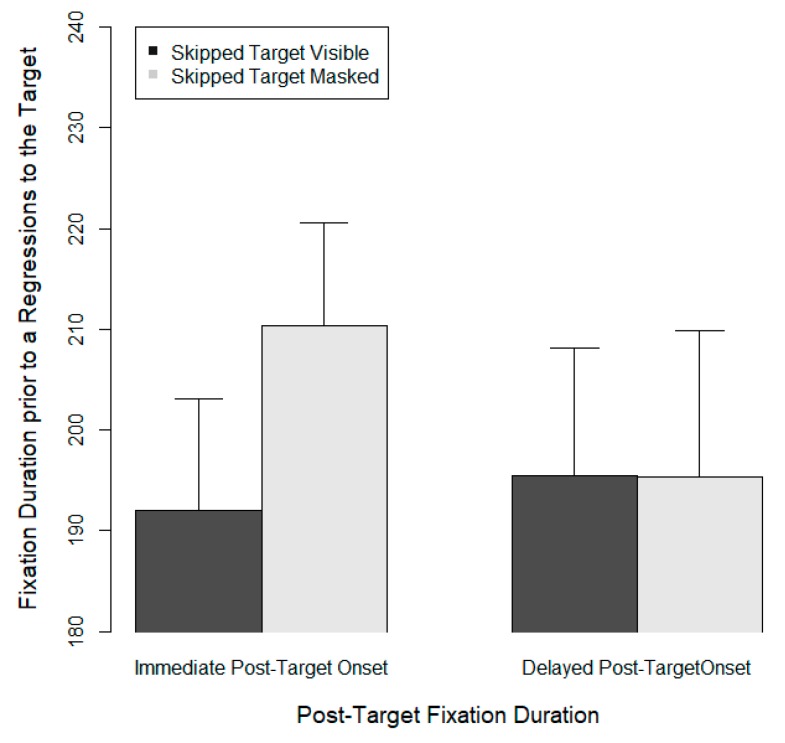
Fixation durations on post-target words prior to a regression to a skipped target word. Fixation durations and standard errors are shown as a function of the visibility of the skipped target and the timeline of post-target onset.

Examination of eye movements showed that target skipping was once more relatively common (951 skips on 2657 trials). Again, only slightly more than half of the target skipping (*n* = 518) occurred when the target had been visible prior to skipping; the remaining skipping occurred when the target was masked. After skipping, there were 248 regressions where the eyes moved back to the target. Figure 3 shows the post-target viewing durations for these cases, that is, when target skipping was followed by a regression back to the target. These post-target viewing durations were examined as a function of the visibility of the target prior to skipping (visible vs. masked) and also as a function of post-target onset, that is, whether the fixated post-target word was visible immediately or whether the onset of useful information was briefly delayed. 

A linear mixed model yielded a relatively small estimated effect of post-target onset on pre-regression fixation duration, less than 4 ms (b = 3.41, SE = 14.20, *t* = 0.24, *p* > 0.7) and no effect to target visibility (*p* > 0.5). Although Figure 3 appears to suggest a moderating influence of the visibility of the skipped target in the immediate post-target onset condition, the corresponding interaction did not approach significance (*p* = 0.36). The main finding, a null effect for post-target onset effect, suggests that readers did not seek to extract linguistic information from the post-target word prior to the programming of a regression to a skipped target. Since the data set was small and null effects are difficult to interpret, this account needs to be considered tentative.

## 4. Individual Differences 

A recent examination of basic oculomotor indexes (fixation duration and saccade size) in four perceptual tasks revealed stable individual differences across tasks and over time [44]. Since eye movements are part and parcel of fluent reading, individuals may also develop characteristic regression strategies. 

Cluster analyses applied to indexes of eye movements during the reading of structurally different story segments [45] are consistent with this view. Clustering of readers yielded three approximately equal-size groups of fluent readers with distinct eye movement preferences. The hallmark of one group was the frequent use of regressions. These readers were assumed to resolve comprehension difficulties through the re-reading of structurally important parts of the story. Members of the two other groups preferred to move the eyes forward in the text, and they may have attempted to resolve difficulties through the reading of upcoming text. 

Similarly, path analyses, that examine the directionality of associated effects [46], yielded a relatively weak link between oculomotor variables that control the encoding of text (skipping rates, the size of forward directed saccades, and fixation duration) and successful reading comprehension. Reprocessing (use of regressions and re-reading time), by contrast, was strongly associated with successful comprehension. In the most effective model, encoding and reprocessing were also strongly linked, suggesting that regressions are an oculomotor tool that can be used to achieve a more accurate representation of linguistic content when the initial encoding of text was poor (i.e., when skipping was common and when viewing durations were short). Cluster analysis (with a three group solution) yielded one group with low comprehension scores. This group consisted of slow readers who regressed rarely. The two other groups achieved a relatively high reading comprehension score: One group consisted of fast readers who regressed rarely, and the other of slower readers who regressed relatively often. With slower readers, it was thus the use of regressions that distinguished readers with good and poor text comprehension.

These analyses suggest that some readers learned to use regressions to improve their text comprehension. Other readers may “under-use” regressions because they have not learned to regress or because the benefit of rereading is not transparent. To gain some insight into the “under-use” of regressions, we examined whether readers’ would regress more often after the benefits of re-reading have become transparent. 

For this, we re-analyzed the results of two experiments [28] in which the identity of a target word was manipulated in one of the experimental conditions so that the execution of a regression to it resolved a processing difficulty when a target was incongruent with prior context. As noted earlier, targets were followed by different sentence context: One in which the target and its subsequent context were congruent, one in which they were incongruent, and one in which their relationship was “neutral”. For instance, the target word birch in “The midwife thought that the birch …” was followed by “was the most beautiful tree in the yard” (congruent), or “was successful despite the mother’s concerns” (incongruent), or “was a strange sight first thing in the morning” (neutral). By design, all targets had an orthographic neighbor, “birth” in the example, that agreed with incongruent context. In the incongruent condition, regression-contingent display changes were implemented that replaced the target with a much better fitting neighbor when readers regressed to the target location. The execution of a regression to an incongruent target was thus rewarded with the viewing of a word, a congruent orthographic neighbor, that resolved the processing difficulty. 

**Figure 4:**
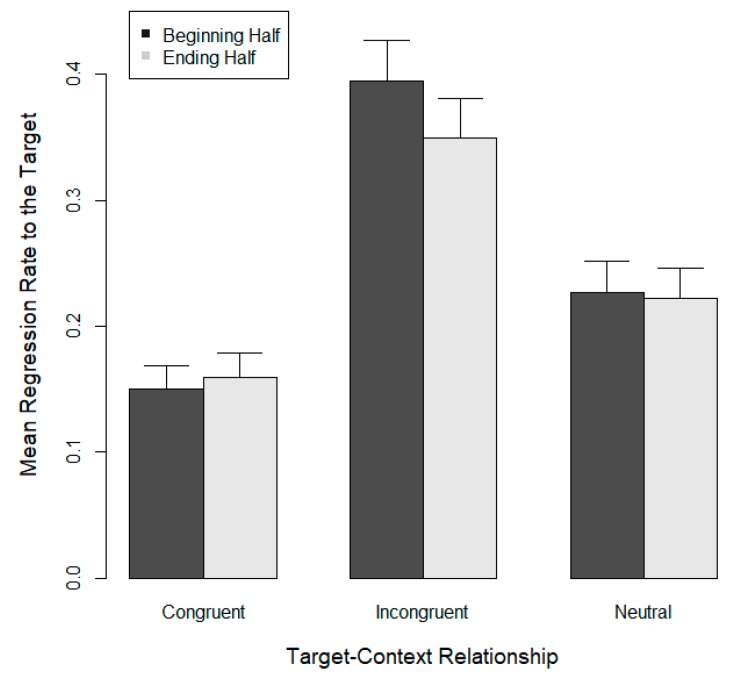
Mean regression rate to target words and standard errors as a function of targets’ consistency with subsequent sentence content. Incongruent target words were replaced with congruent targets when readers regressed back to the target.

Regressions to targets during the first half of the trials were compared with regressions to targets during the second half, the assumption being that the usefulness of regressions in the incongruent condition would increase their occurrence over the course of the experiment. No change in regression frequency, or a lesser change, was expected for the congruent and the neutral conditions. Figure 4 shows regression rates to targets as a function of trial sequence (beginning vs. ending half) and sentence context (74 participants who participated in Experiments 1 and 2 [28]). As can be seen, regression rates did not increase over the course of the experiment in the incongruent condition, and the interaction of trial sequence with context was negligible (*p* > 0.3).

These results indicate that readers under-use regressions even when the benefits of rereading are relatively transparent.

## 5. Regressions and the Teaching of Reading

Why did readers’ use of regressions not increase over the course of the experiments in the incongruent condition? Some readers may have under-used large regressions because they were content with a somewhat odd—but “good-enough”—representation of sentence meaning [28]. Other readers may not have learned to use regressions strategically. In a large scale (*n* = 632) study of children’s eye movements during reading [47], increases in reading skill from grades one to five were associated with the execution of fewer and larger saccades and the use of shorter fixation durations. However, the proportion of inter-word regressions increased only after grade four. From then on, more skillful readers “may go back to words that might have caused processing difficulty” [47] (p. 477). Even older readers may not acquire this strategic skill without explicit instruction. 

Many readers may also prefer to resolve difficulties through a reprocessing of working memory content [31]. For instance, they could “normalize” the representation of sentence meaning by assuming that a represented word form was misperceived, or, as mentioned before, they could be content with a somewhat odd sentence meaning [48,49,50]. Current reading instruction emphasizes forward-directed reading, as this is necessary for successful text comprehension. Regressions could even be discouraged, as they interrupt the speech-like forward-directed flow of information uptake. This is particularly likely in the case of reading aloud, which is the predominant form of reading in lower grades, as regressions for meaning will likely compromise eye-voice-coordination [48]. Oculomotor studies of reading development indicate that there is a transition from a more sequential and sub-lexical beginning stage towards lexical reading, which appears common at about grade four [44]. More generally, large-scale psychometric analyses of individual differences in reading development indicate a systematic increase in the variance accounted for by higher order comprehension at the expense of decoding [49]. As this development progresses, it may be beneficial to systematically teach the use of re-inspection strategies when comprehension is suboptimal. Indeed, successful developing readers routinely engage in the monitoring of comprehension and use regressions to resolve inconsistencies [50]. As we have argued above, this skill may not develop automatically and should therefore become a more explicit focus of reading instruction. 

## 6. Conclusions

Reading differs from spoken language processing in that linguistic symbols have spatial properties that are relatively stable. The forms of depicted words thus remain accessible after they have been identified, and some readers discover that regressions to prior text can help resolve word recognition and text comprehension difficulties during silent reading. Somewhat surprisingly, supplementary analyses of pertinent data suggest that even skilled readers do not take full advantage of this opportunity.
